# Intraoperative Challenges of Patient-Specific Osteotomy Guides in Anterior Maxillary Osteotomy: A Case Report

**DOI:** 10.7759/cureus.115725

**Published:** 2026-09-03

**Authors:** A. Saneem Ahamed, Sri Devi, N. S. Rajessh, Jeeva Shri, Nandhini Sankaran

**Affiliations:** 1 Department of Oral and Maxillofacial Surgery, Priyadarshini Dental College and Hospital, Thiruvallur, IND

**Keywords:** anterior maxillary osteotomy, cad/cam, maxillary prognathism, orthognathic surgery, surgical guides, two-piece osteotomy, virtual surgical planning

## Abstract

Orthognathic surgery has increasingly incorporated digital workflows and patient-specific surgical guides to enhance accuracy and predictability. While these technologies offer improved planning and execution, their intraoperative application in segmented maxillary osteotomies remains technically demanding. Limited literature exists describing the practical challenges encountered during guide-assisted osteotomy, particularly in anatomically constrained regions. This case report describes the use of a patient-specific cutting guide in a 24-year-old male patient undergoing anterior two-piece maxillary osteotomy for correction of skeletal Class II malocclusion with gummy smile. Virtual surgical planning was performed using cone-beam computed tomography and digital dental models to simulate a 7 mm maxillary setback and 4 mm superior impaction. A custom osteotomy guide and surgical splint were fabricated using computer-aided design (CAD)/computer-aided manufacturing (CAM) technology. Intraoperatively, the guide was positioned following mucoperiosteal flap elevation and used to perform vertical and horizontal osteotomy cuts. The maxillary segment was mobilized, repositioned using a prefabricated occlusal splint, and stabilized with titanium miniplates. The use of the osteotomy guide facilitated accurate transfer of the planned osteotomy lines and segment repositioning. However, intraoperative challenges were encountered, including increased operative time due to guide positioning and verification, limited access during osteotomy cuts, and difficulty in executing palatal osteotomy. Initial instability of the guide due to soft tissue interference required intraoperative adjustment. A minor palatal mucosal tear occurred, which was managed conservatively. Despite these challenges, satisfactory occlusal alignment and postoperative stability were achieved.

## Introduction

Anterior maxillary osteotomy (AMO) is a well-established orthognathic procedure used to correct dentofacial deformities such as maxillary protrusion, vertical maxillary excess, and gummy smile. The procedure involves creating controlled osteotomies in the anterior maxilla to mobilize and reposition the dentoalveolar segment according to the planned correction. In segmented or two-piece procedures, the maxillary dentoalveolar segment is divided into separate portions, making accurate localization of the osteotomy cuts and controlled segment positioning particularly important. Precise execution of the osteotomy and accurate repositioning of the maxillary segment are critical to achieving the planned functional and esthetic outcomes [1]. Traditionally, these procedures have relied on model surgery and occlusal splints, which are technique-sensitive and may be subject to cumulative errors during laboratory and intraoperative transfer.

The advent of virtual surgical planning (VSP) and computer-aided design (CAD)/computer-aided manufacturing (CAM) technology has transformed orthognathic surgery by enabling three-dimensional (3D) planning and fabrication of patient-specific surgical guides. These guides are customized templates designed from the patient's 3D anatomy and virtual surgical plan and are positioned intraoperatively on the dentition or supporting anatomical structures to indicate the planned osteotomy location and facilitate transfer of the virtual plan to the surgical field [2]. In anterior maxillary osteotomy, where interdental osteotomies and controlled segment movement are required, patient-specific surgical guides may facilitate more precise and reproducible osteotomy positioning [1].

Despite these advantages, the intraoperative use of patient-specific osteotomy guides presents several technical challenges. Factors such as limited surgical access, difficulty in guide adaptation due to soft tissue interference, restricted instrument maneuverability, and challenges in performing palatal osteotomy can complicate the procedure [3]. Additionally, guide stabilization and accurate seating are critical for ensuring fidelity to the planned osteotomy, and any deviation may compromise surgical outcomes. These challenges may be further compounded during the initial adoption phase as clinicians become familiar with virtual planning and computer-assisted surgical workflows [2].

While previous studies have primarily evaluated the accuracy and reproducibility of computer-assisted orthognathic surgery, limited clinical reporting has focused on the practical intraoperative challenges encountered during patient-specific guide-assisted anterior maxillary osteotomy, including guide stabilization, surgical access, and execution of interdental and palatal osteotomies [4,5]. Understanding these technical challenges may help optimize intraoperative guide seating and stabilization, facilitate controlled interdental and palatal osteotomies, and minimize intraoperative modifications during anterior maxillary osteotomy.

This case report aims to describe the use of a patient-specific osteotomy guide in anterior maxillary osteotomy and to highlight the intraoperative challenges encountered during its application.

## Case presentation

A 24-year-old male patient presented with a chief complaint of forwardly placed upper anterior teeth and an unaesthetic smile. The patient had undergone 1.5 years of comprehensive fixed orthodontic treatment and remained in active fixed orthodontic appliances.

Clinical examination revealed maxillary protrusion, and AMO was planned as part of comprehensive orthodontic-surgical treatment. Preoperative VSP was performed by integrating CBCT data with digital dental models to plan the osteotomy and repositioning of the anterior maxillary segment (Figure 1).

**Figure 1:**
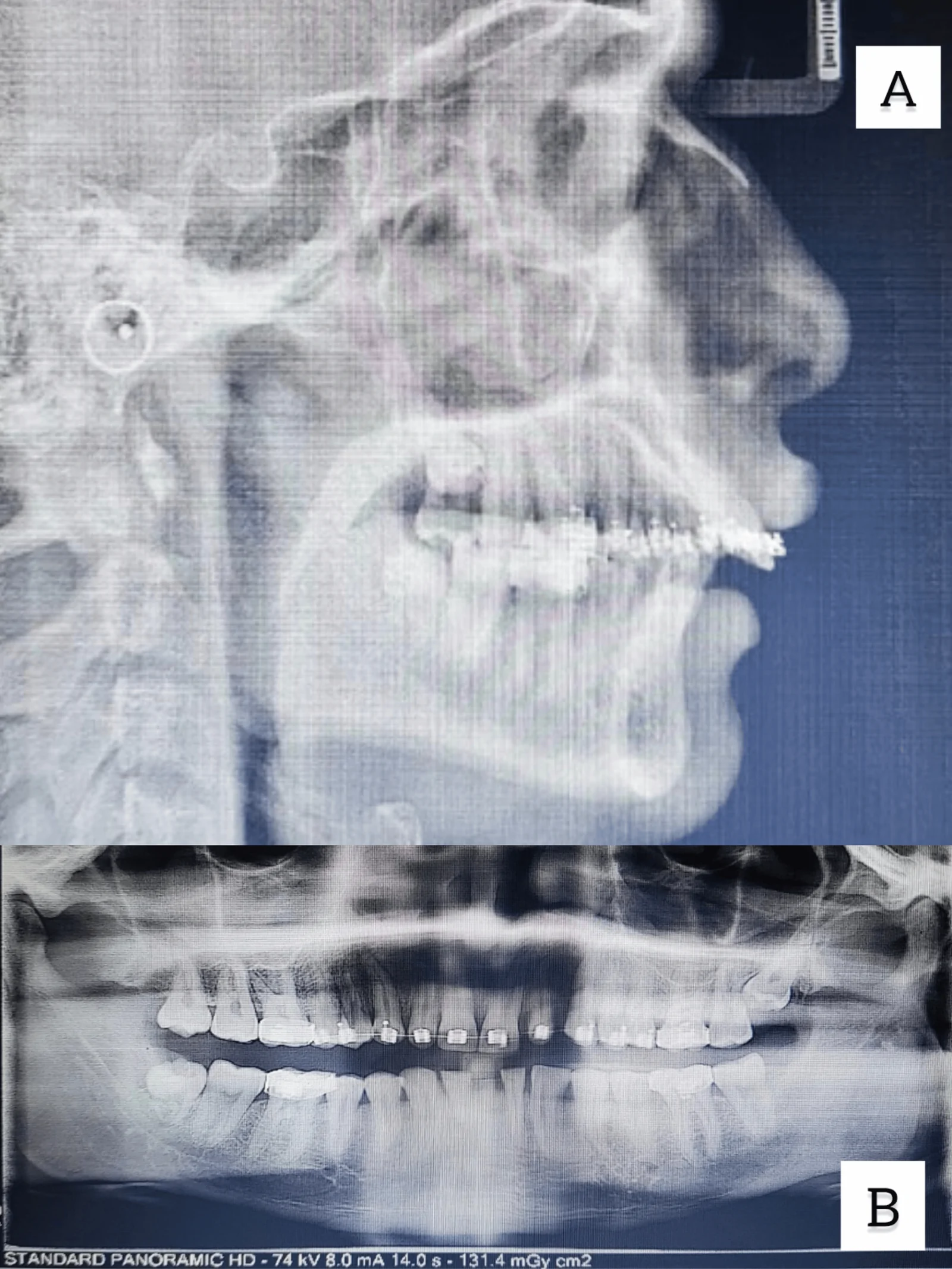
Preoperative (A) lateral cephalogram and (B) orthopantomogram

The planned AMO, setback, and superior impaction were virtually simulated, following which a patient-specific AMO cutting guide was designed using CAD software and fabricated using 3D printing with surgical guide resin (Figure 2). A 3D composite model was generated, and osteotomy lines were simulated with a planned 7 mm maxillary setback and 4 mm superior impaction. Special attention was given to the inter-dental osteotomy sites to maintain a safe distance from adjacent tooth roots.

**Figure 2:**
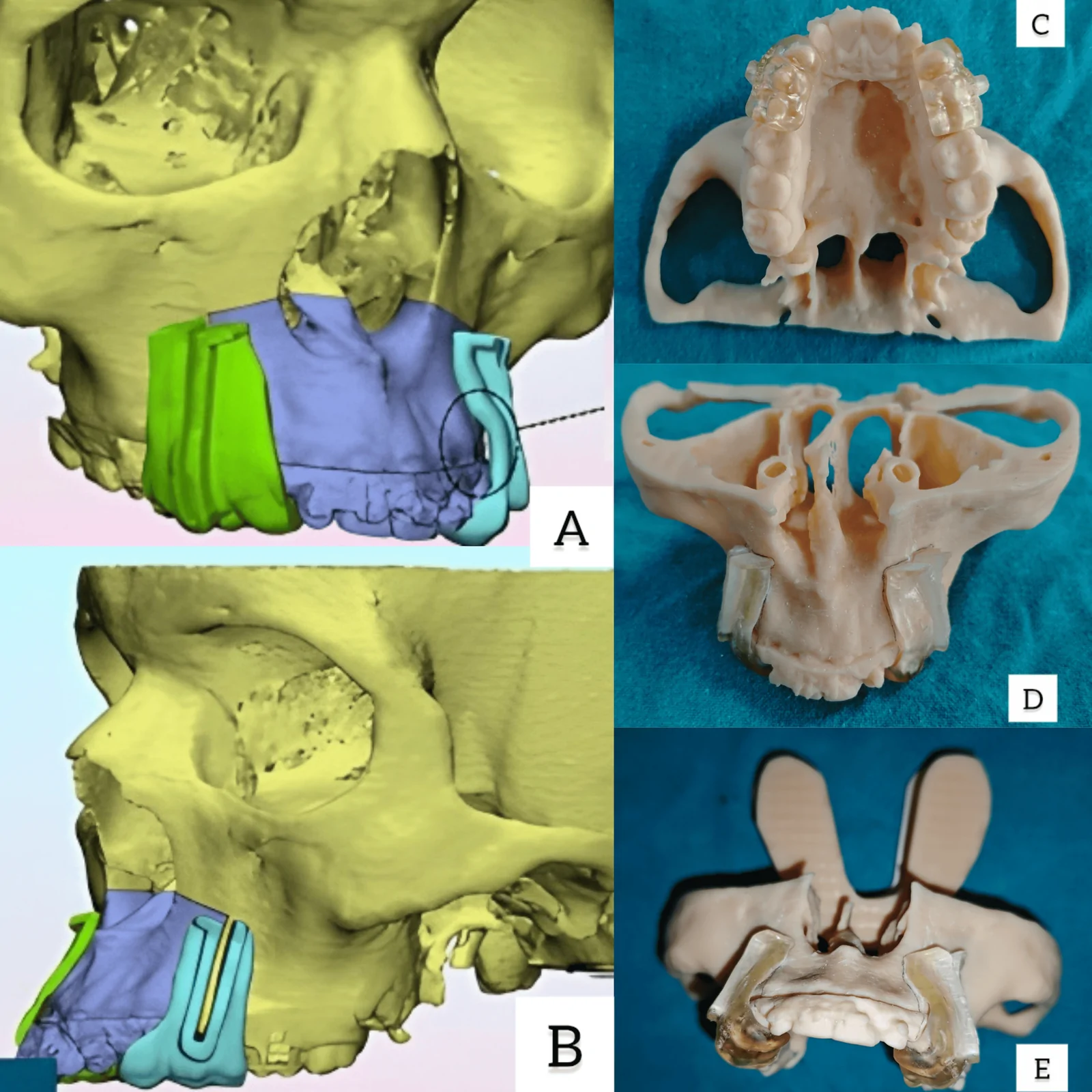
Design and fabrication of the patient-specific anterior maxillary osteotomy guide. (A) Frontal VSP model with the osteotomy guide. (B) Lateral VSP model demonstrating guide positioning. (C) Occlusal view of the fabricated 3D-printed guide. (D) Inferior view of the printed guide showing the fitting surface. (E) Final patient-specific guide ready for surgical application. The VSP images use color coding to distinguish the anterior maxillary dentoalveolar segment from the adjacent maxillary structures and the patient-specific guide, facilitating visualization of the planned osteotomy and guide position. VSP: virtual surgical planning; 3D: three-dimensional

Under general anesthesia, local infiltration with a vasoconstrictor was administered. A maxillary vestibular incision was placed, and a full-thickness mucoperiosteal flap was elevated to expose the anterior maxilla (Figure 3). Premolar extractions were performed as planned to facilitate osteotomy and segment repositioning.

**Figure 3:**
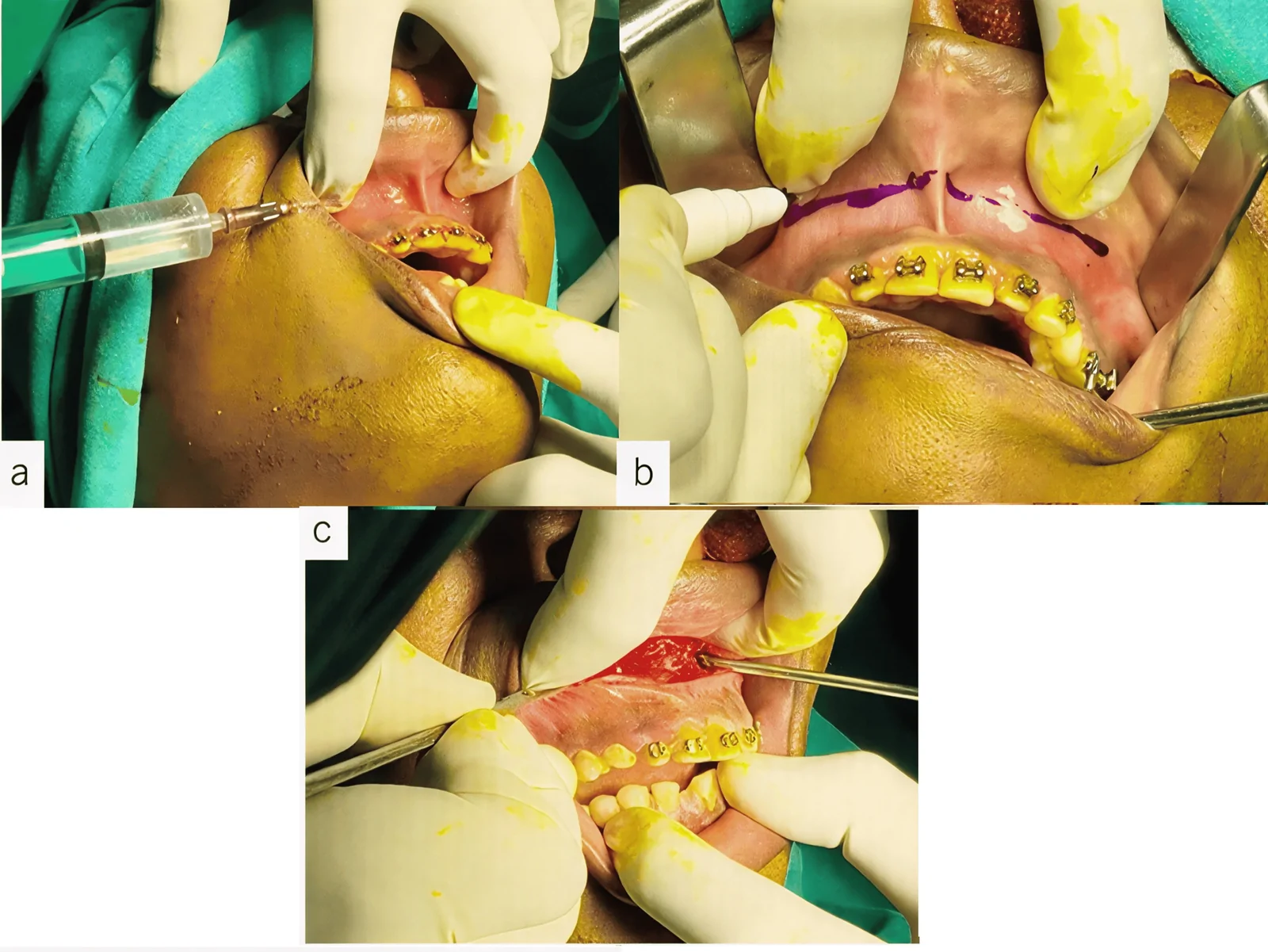
Intraoperative images (a) Local anesthetic with vasoconstrictor administered; (b) marking of incision done; (c) incision placed

Initial difficulty was encountered in achieving passive seating of the guide due to soft tissue interference and minor discrepancies in adaptation, requiring additional flap reflection and intraoperative adjustments. Once stabilized, the guide was seated using a tooth-supported friction fit without additional fixation pins or screws, and osteotomy cuts were performed along the predefined slots. Limited working space and restricted instrument angulation were noted during the execution of vertical and horizontal cuts, necessitating careful manipulation of surgical instruments.

Following completion of the buccal osteotomy, palatal osteotomy was performed with caution. As the guide did not provide direct assistance in the palatal region, this step required conventional surgical technique. A minor palatal mucosal tear occurred during this phase, which was managed conservatively. The osteotomized segment was mobilized (down-fractured) and repositioned using the prefabricated occlusal splint, followed by intermaxillary fixation to achieve the planned occlusion (Figure 4).

**Figure 4:**
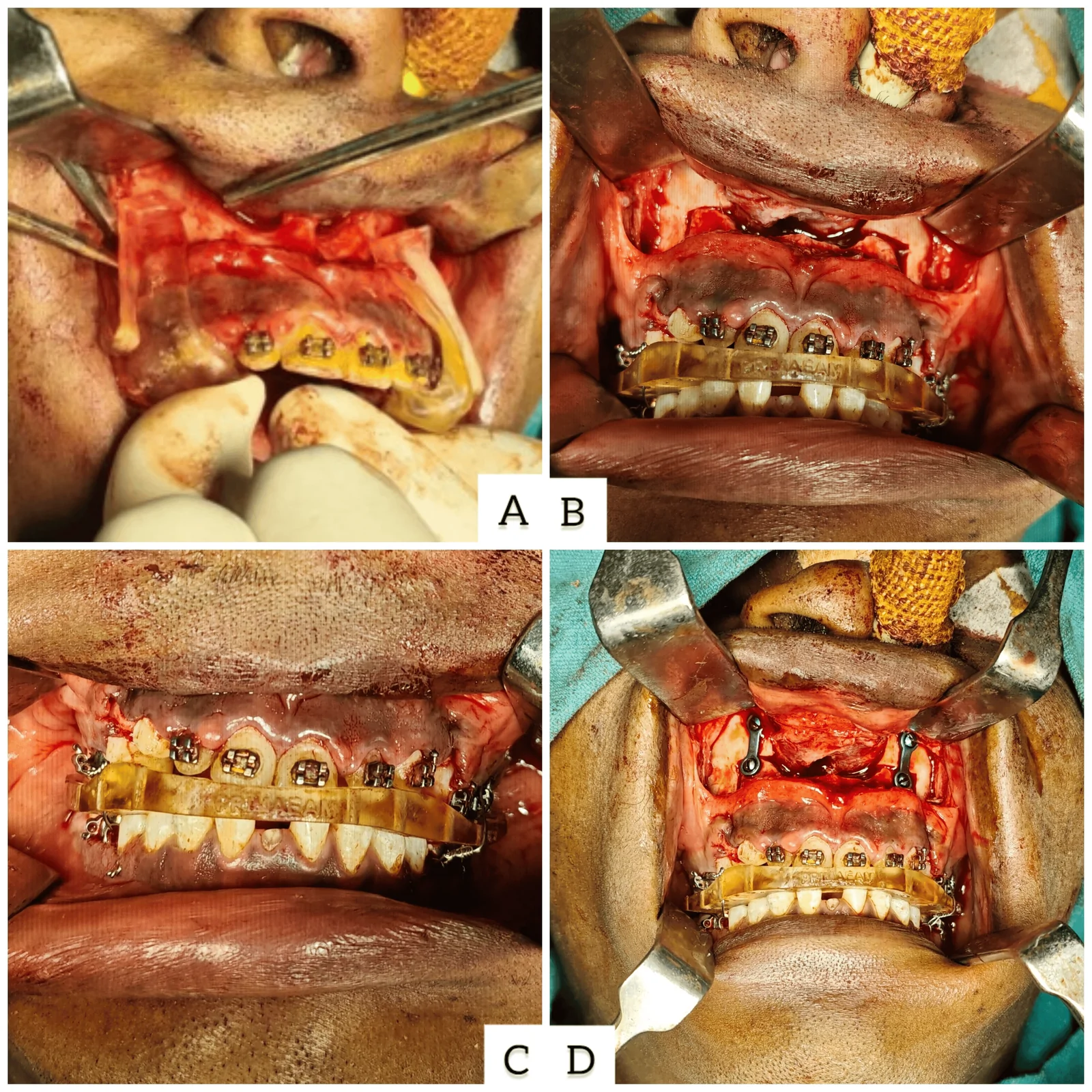
Intraoperative application of the patient-specific osteotomy guide and fixation (A) Placement and stabilization of the patient-specific osteotomy guide. (B) Verification of guide fit and occlusal positioning with the intermediate splint. (C) Repositioned anterior maxillary segment maintained in the planned position with the occlusal splint. (D) Definitive fixation of the osteotomized segment using titanium miniplates and screws after guide removal.

Rigid internal fixation was achieved using titanium miniplates and screws. Hemostasis was ensured, and the surgical site was closed in layers. At the one-month postoperative follow-up, clinical evaluation demonstrated satisfactory occlusion and stable positioning of the anterior maxillary segment (Figure 5).

**Figure 5:**
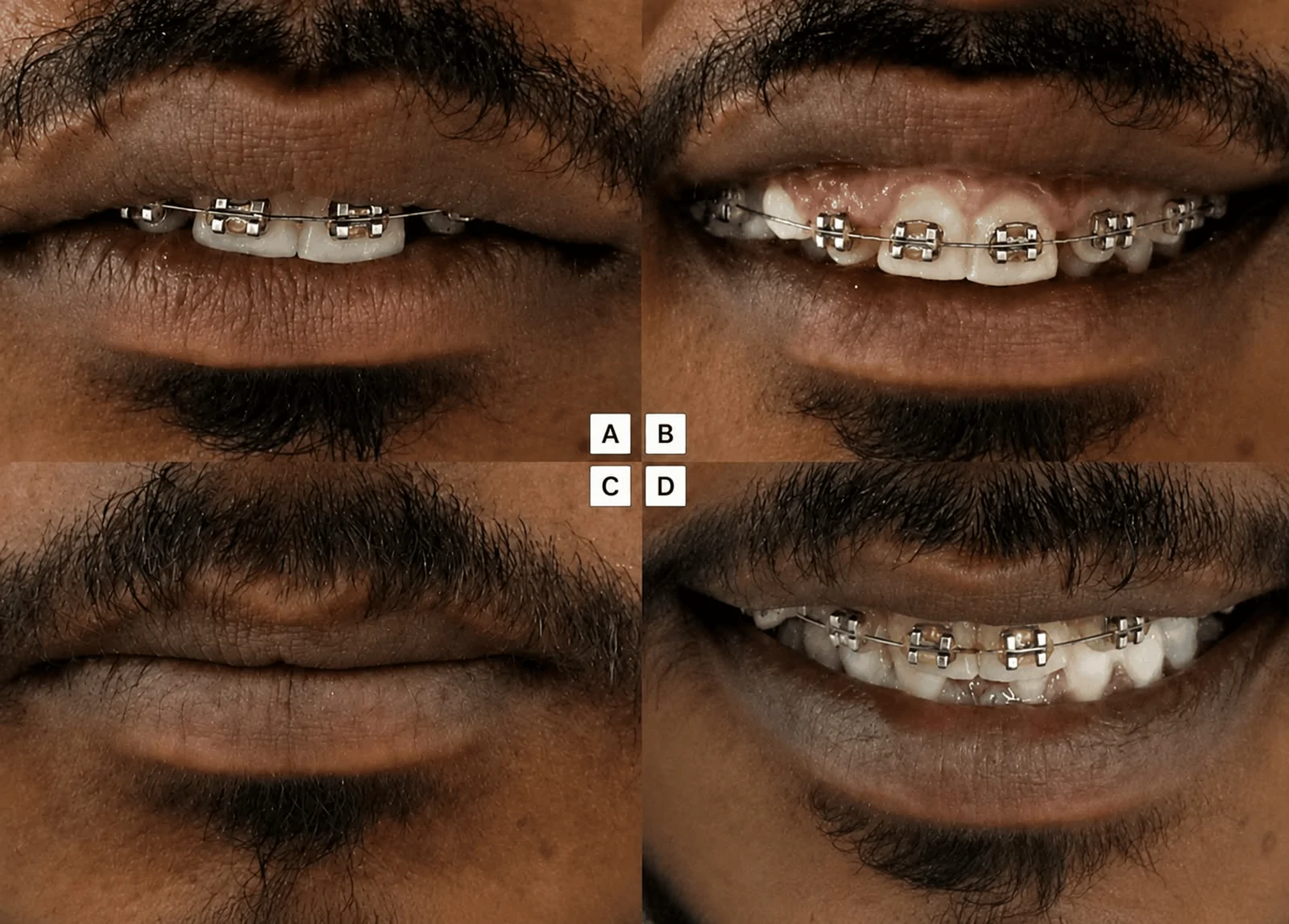
Preoperative and postoperative extraoral comparison. (A) Preoperative lips at rest. (B) Preoperative smile. (C) Postoperative lips at rest. (D) Postoperative smile showing improved lip competence and reduced gingival display.

## Discussion

The incorporation of patient-specific surgical guides into virtual surgical planning has been associated with improved accuracy in transferring planned skeletal movements to the surgical field. A recent systematic review and meta-analysis demonstrated a 1.20-mm reduction in the discrepancy between planned and actual jaw position when VSP incorporating custom surgical guides was compared with conventional planning. However, their intraoperative application, particularly in AMO, presents several practical challenges [6]. This case report highlights key difficulties encountered during guide-assisted AMO, emphasizing operative time, osteotomy execution, palatal osteotomy, guide stabilization, and cost considerations.

Operative time

The total operative time from incision to completion was 4.5 hours (270 minutes). Guide positioning, passive-fit verification, and minor adjustments contributed to the initial operative period; however, once satisfactory seating was achieved, the guide facilitated transfer of the planned osteotomy. Previous studies have reported variable effects of patient-specific surgical guides on operative time, with a recent systematic review and meta-analysis finding a potential reduction in operative time with VSP and custom guides, although the overall difference was not statistically significant [6].

Osteotomy difficulty

Guide-assisted osteotomy improved the accuracy of cut placement but introduced challenges related to restricted surgical access. The physical bulk of the guide and predefined cutting slots limited the angulation and maneuverability of rotary instruments. This was particularly evident during vertical and horizontal osteotomy cuts in the anterior maxilla, where working space is inherently limited. Additionally, reliance on guide-defined pathways reduced tactile feedback, requiring careful handling to ensure complete and controlled bone cuts [7]. Despite these challenges, the guide provided a significant advantage in avoiding root injury and maintaining planned osteotomy levels.

Palatal osteotomy difficulty

Palatal osteotomy remained the most technically challenging step in the procedure. The surgical guide primarily facilitated buccal osteotomy cuts and did not provide direct assistance for palatal bone separation. Consequently, this step required conventional freehand technique with limited visibility and access. The lack of guide support increased the risk of complications, as evidenced by the occurrence of a minor palatal mucosal tear in this case. This highlights a key limitation of current guide designs, emphasizing the need for surgical expertise and caution during palatal execution [8].

Guide stabilization

Accurate seating and stabilization of the osteotomy guide are critical for ensuring fidelity to the VSP. In this case, initial difficulty was encountered due to soft tissue interference and minor discrepancies in guide adaptation. Additional flap reflection and bone surface preparation were required to achieve stable positioning. Any instability or micromovement of the guide can compromise the accuracy of osteotomy cuts, making this a crucial step in the workflow [9]. Proper preoperative design and meticulous intraoperative handling are essential to ensure optimal guide performance.

Cost considerations

The use of a patient-specific osteotomy guide involved additional expenditure related to VSP and 3D guide fabrication. In the present case, the custom AMO cutting guide and associated maxillary model were fabricated using surgical guide resin. Although patient-specific guides involve additional upfront costs, their potential clinical value lies in facilitating accurate transfer of the VSP and simplifying intraoperative execution. However, a formal cost-effectiveness assessment was beyond the scope of this single case report [10].

The principal limitation of this report was the absence of postoperative CBCT, which precluded 3D superimposition and objective quantification of the discrepancy between the planned and achieved 7-mm setback and 4-mm superior impaction. Assessment was therefore limited to postoperative clinical findings and orthopantomographic evaluation

## Conclusions

Patient-specific osteotomy guides in AMO enhance surgical precision and facilitate accurate transfer of virtual planning to the operative field. However, their use is associated with technical challenges, including increased operative time, restricted access during osteotomy, difficulty in palatal execution, and the need for precise guide stabilization. Despite these limitations, with adequate surgical expertise and familiarity with digital workflows, these challenges can be effectively managed. Overall, osteotomy guides represent a valuable adjunct in orthognathic surgery, offering improved predictability and potential long-term benefits in clinical outcomes.
